# Characterization of Thick and Thin Film SiCN for Pressure Sensing at High Temperatures

**DOI:** 10.3390/s100201338

**Published:** 2010-02-11

**Authors:** Alfin Leo, Sergey Andronenko, Ion Stiharu, Rama B. Bhat

**Affiliations:** 1 CONCAVE Research Centre, Department of Mechanical and Industrial Engineering, Concordia University, Montreal, Quebec H3G 1M8, Canada; E-Mail: alfinleo@gmail.com; sergey.andronenko@gmail.com; 2 Department of Mechanical and Industrial Engineering, Concordia University, Montreal, Quebec H3G 1M8, Canada; E-Mail: rbhat@encs.concordia.ca

**Keywords:** SiCN, polymer derived ceramic, dynamic pressure sensing at high temperatures

## Abstract

Pressure measurement in high temperature environments is important in many applications to provide valuable information for performance studies. Information on pressure patterns is highly desirable for improving performance, condition monitoring and accurate prediction of the remaining life of systems that operate in extremely high temperature environments, such as gas turbine engines. A number of technologies have been recently investigated, however these technologies target specific applications and they are limited by the maximum operating temperature. Thick and thin films of SiCN can withstand high temperatures. SiCN is a polymer-derived ceramic with liquid phase polymer as its starting material. This provides the advantage that it can be molded to any shape. CERASET™ also yields itself for photolithography, with the addition of photo initiator 2, 2-Dimethoxy-2-phenyl-acetophenone (DMPA), thereby enabling photolithographical patterning of the pre-ceramic polymer using UV lithography. SiCN fabrication includes thermosetting, crosslinking and pyrolysis. The technology is still under investigation for stability and improved performance. This work presents the preparation of SiCN films to be used as the body of a sensor for pressure measurements in high temperature environments. The sensor employs the phenomenon of drag effect. The pressure sensor consists of a slender sensitive element and a thick blocking element. The dimensions and thickness of the films depend on the intended application of the sensors. Fabrication methods of SiCN ceramics both as thin (about 40–60 μm) and thick (about 2–3 mm) films for high temperature applications are discussed. In addition, the influence of thermosetting and annealing processes on mechanical properties is investigated.

## 1. Introduction

Gas turbine engines in the aerospace industry operate at very challenging levels of performance requiring stable operation of the compressor at all times. However, during take off and landing, transient pressure fluctuations normally arise, resulting in blade stall that is propagated to adjacent blades and back through the various stages of the compressor. This could lead to engine surge for high speed turbines in fraction of seconds and subsequently damage the engine [1].

Blade stall generally occurs as a result of unsteady pressure fluctuations, resulting in a tendency for flow to separate, which creates a constricted passage for further incoming air. As a result, reverse flow conditions are established and propagated back through the engine, giving rise to compressor surge. Complete avoidance of the initiation of conditions that will lead to surge or stall, requires determination of operational margins that are far from the stability limit of the compression system. Weigl *et al.* [2] demonstrated that stable operation could be achieved over a wider range of flow conditions with a minimal loss in performance through active or passive control of the flow conditions.

In order to avoid such catastrophic instabilities, it is imperative to have a reliable control system for early detection of incipient surge. However, the effective implementation requires the use of sensors, actuators and controllers to detect and rectify the incipient instabilities of the system. The widely used semiconductor pressure sensors capable of operating at high temperature by Kulite semiconductors Inc and Vibro-meter have several limitations including reduced long-term stability and reliability. These include limited time operation, very limited reliability after stipulated minutes of operation and severe drift to temperature changes.

In addition to the above limitations, these sensors are bulky, thereby limiting their use for online condition monitoring, accompanied by a significant increase in weight. This would consequently add to the cost offsetting any benefits derived from an enhanced engine performance. Hence it is desirable to use micro transducers for dynamic pressure measurements located on the stators of the various stages of the compressor.

MEMS sensors are mostly based on silicon as the sensing material as well as substrate for the micro circuitry, which restricts its use below 200 °C. In order to overcome this drawback, extensive research is currently in progress to use Silicon Carbide MEMS technology (SiC-MEMS). Sensors fabricated in Silicon On Insulator (SOI) and silicon carbide (SiC) have been reported and they are rated to be functional in a temperature range of 200 °C and 400 °C, respectively [3,4].

However, the processes associated with SiC-MEMS have been known to be time consuming, expensive, technically very challenging and are limited for use below 500 °C. Ceramics could be used to sense temperatures above 500 °C. However, there are challenges in preparing them to the desired shape and thickness.

Quest for a sensor to operate in a temperature range of more than 500 °C and to also address the fabrication issues resulted in the exploitation of a new class of polymer-derived ceramics, which essentially consist of amorphous alloys of silicon, carbon and nitrogen, and is known as Silicon Carbon Nitride (SiCN). The service temperature of SiCN is expected to be in the range of 1,400 °C, and could be originally shaped into any form as a thermoset polymer [5].

In this study, methods for shaping of polymer-derived SiCN ceramics into thin and thick films are described. Furthermore, the influence of parameters such as thermosetting and annealing temperatures on the properties of SiCN ceramics including hardness and reduced Young’s modulus, which are important for the construction of pressure sensors, are discussed.

## 2. Sensing Scheme Overview

**Figure 1.**
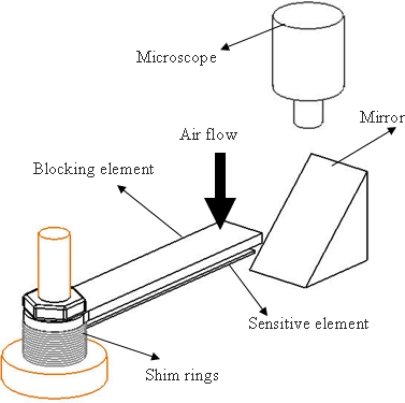
The experimental schematic for dynamic pressure sensing [8].

The need for a sensor for high temperature applications is emphasized by Andronenko *et al.* [6]. They proposed a hybrid processing method by embedding piezoresistive chromium strain gauge between two thin SiCN membranes for sensing pressure in high temperature environments. MEMS-based flow direction and flow rate sensor based on eight curved cantilever beams arranged evenly in an octagonal manner is proposed by Lee *et al.* [7], where the air flow on the upper surface of the beam makes it deflect proportional to the flow. Leo *et al.* [8] have demonstrated experimentally a novel method for pressure sensing using an indirect drag effect. The sensor has a blocking element and a sensitive element. The function of the blocking element is to protect the slender sensitive elements against direct impingement of the flow. Hence, it is mandatory that the blocking element is thicker (as the blocking element withstands the force exerted by the flowing fluid) than the sensitive element (as thickness of the sensitive element determines the sensitivity of the sensor). The sensitive element senses the pressure drop caused by the blocking element and relates that to the input flow velocity/dynamic pressure. Figure 1 shows the schematic diagram of the experimental setup (in macro scale using an aluminum sensitive element and steel blocking element) that was used to measure the dynamic pressure at room temperature conditions [8]. The air flow past the blocking element creates a pressure drop behind the blocking element. With the increase in the velocity of the air this pressure drop increases to a level wherein the sensitive element deflects towards the blocking element. This deflection could possibly be correlated to the air flow velocity or in turn dynamic pressure.

The image of the tip of the cantilever sensitive element is reflected through the mirror (which does not produce any magnification) and deflection was measured using a microscope (with a graduated eye piece). The gap between the blocking element and the sensitive element was maintained using a shim ring (of thickness ranging from 300–500 microns)

**Figure 2.**
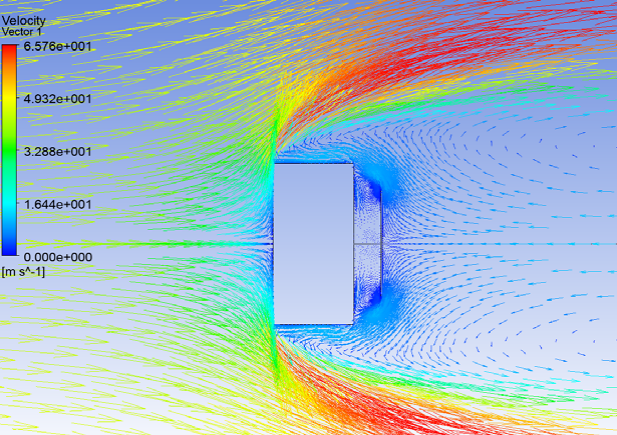
Velocity vectors close to the blocking / sensitive element.

A numerical simulation was performed to understand the phenomenon using ANSYS CFX. The velocity profile of the flow pattern behind the sensitive element for an input velocity of 50 m/s directed from left to right is shown in Figure 2.

From Figure 2 the deflection of the sensitive element is due to

❖ The stream of vortex that hits back the sensitive element
❖ The suction of air between the blocking and the sensitive element when the stream of vortex passes the gap.

A detailed numerical analysis of the phenomenon requires two-way fluid structure interactions. The numerical simulation requires transfer of fluid load from the fluid domain to a structural domain and then again the displacement has to be transferred from structural displacement to the fluid domain. This two-way fluid structure interaction is available in the latest version of ANSYS released recently. A more detailed description of the sensor and its performance are detailed in [8] based on experimental investigation.

The same phenomenon could be extended at high temperature as the working principle of the sensor is not limited by the temperature level. However the blocking and sensitive element has to be made up of special material like SiCN to withstand high temperature. The elastic properties of the material are expected to change at high temperature. Silicon carbon nitride (SiCN) is the material of choice as it withstands temperature in the range of 1,400 °C. The current study focuses on the fabrication of the proposed sensor.

## 3. State of the Art

Silicon carbon nitrate (SiCN) is a polymer-derived ceramic (PCD), which is a new class of ceramics derived from liquid precursors, called polysilazanes. The processing route for SiCN is micro casting that favors the fabrication of low cost, mass fabrication of such MEMS devices [9]. The process consists of the following steps:

Thermal Setting: The liquid precursor is cast into a mold of desired shape and undergoes thermal treatment (with or without thermal initiator) to create a rigid polymer.
Crosslinking: The rigid polymer is crosslinked at higher temperature and pressure. Crosslinking creates bonds that link one polymer chain to another.
Pyrolysis: The free standing forms are pyrolyzed at 400–1,100 °C under a controlled atmosphere yielding SiCN at 1,000 °C.

SiCN is suggested for high temperature applications mainly because of its oxidization resistance at high temperature. The oxidation behavior of SiCN is studied at temperature ranges of 900 °C–1,200 °C [10], and the results suggest that at these temperatures the oxidation of SiCN is similar to that of Silicon Carbide (SiC), in spite of the presence of nitrogen in SiCN. The oxidation resistance of SiCN resulted in exploration of new type of PCD called silicoaluminum carbonitride (SiAlCN). SiAlCN possesses high resistance to oxidation and high corrosion due to the presence of aluminum in the oxide layer that is formed [11]. Polyaluminasilazanes were used as the precursor for SiAlCN and tested for oxidation resistance up to 1,400 °C [12]. The oxidation of SiCN is similar to SiAlCN until 900 °C, however at higher temperature the oxidation rates of SiAlCN decrease with annealing time [13].

The electrical properties possessed by PCDs are also an important factor that PCDs could be used as body of the sensor. The addition of thermal initiator has significant effect on electrical conductivity which first increases and then decreases with increasing concentration of thermal initiator [14]. SiCN ceramic when doped with boron leads to p type conductivity which could be dramatically increased by annealing treatments [15,16]. Addition of manganese powder results in SiCN–Mn which has magnetic properties [17]. SiCN also exhibits piezoresistive behavior with piezoresistive coefficient of ∼1,000–4,000 which is much higher than that of any existing ceramic [18]. It is also observed that the resistivity of SiCN decreases steeply with the applied stress (0–2 MPa) and then decreases slowly with higher applied stress (2–8 MPa) [19].

These characteristics of SiCN make it a valuable material for sensing at high temperature and this paper outlines the fabrication of SiCN as thick and thin films which could be eventually used as a body of the pressure sensor.

## 4. Silicon Carbon-Nitride (SiCN) as Sensor Material

Silicon carbon-nitride (SiCN) was first identified as a coating on silicon substrate by radio frequency sputtering [20]. SiCN compound has aroused a great interest due to its widespread band gap and good field emission characteristics. Also, they exhibit many physical properties comparable to those of diamond due to their short bond length and high bond strength. Chen *et al.* [20] studied the hardness value and effective modulus of crystalline and amorphous SiCN coatings deposited by microwave-enhanced chemical vapor deposition and electron cyclotron resonance plasma CVD, respectively. They reported the hardness value of around 30 GPa for crystalline SiCN and 22 GPa for its amorphous counterpart [20,21].

The excellent physical properties of SiCN and its stability at high temperatures (even at oxidizing atmosphere) and the possibility of making SiCN ceramic from liquid or powder precursor [21], are motivations to use SiCN based solid structures for corrosive and high temperature applications. SiCN ceramic does not oxidize at higher temperature and hence it does not change dimensions due to scale formation and scale shedding.

There is a void in the domain of sensing in high temperature environment (temperature higher than 500 °C) or corrosive environment applications such as gas turbine engine application. This is because most of the MEMS sensing materials are not stable at temperature above 500 °C.

### 4.1. Fabrication of SiCN

Silicon carbon-nitride (SiCN) is a class of recently derived ceramics that remain mechanically stable at temperatures up to 1500 °C in N_2_ and Ar environment, and up to 1,600 °C in air, as well as in corrosive environments [5]. Fabrication of SiCN requires thermosetting, crosslinking and pyrolysis. Thermosetting is performed in the temperature range of 200–240 °C, but the thermosetting temperature can be dramatically reduced to a range of 90–150 °C by adding a catalyst with free radical initiator (Dicumyl peroxide). Crosslinking involves a temperature range of 400 °C–700 °C and pyrolysis is carried at 1,000 °C [5].

SiCN can be made from two different precursors, liquid and powder precursor. However SiCN developed from liquid precursor has shown superior properties [5] than its powder counterpart. The liquid precursor is widely used as it integrates easily to the existing fabrication techniques.

SiCN can be fabricated using two main different techniques, soft lithography and micro molding. These techniques provide solid thermally set polymer, which has to undergo crosslinking and pyrolysis.

The advantage of SiCN in terms of fabrication is that it can be formed to any shape because it is molded in liquid shape, whereas the disadvantage would be the stress induced during the joining process. Attempts have been made to reduce cracks formed due to stress [23] and it is suggested to use fillers and prepyrolysis of the precursor to reduce the stress developed. The PDMS mold is made reusable by spreading an aluminum sheet, unlike the non reusable PDMS mold [23] to release the thermally set polymer. This also reduces the generation of the crack after thermosetting.

It is important to note that SiCN ceramic experiences large shrinkage (up to 38%) during crosslinking and pyrolysis. SiCN ceramics should be separated from substrate or mold before pyrolysis in order to avoid cracks by thermal mismatch. Fabrication techniques and silicon carbon-nitride (SiCN) properties have been studied extensively and it has been found that annealing conditions have impact on their properties [24,25].

#### 4.1.1. Thin Film SiCN

Thin film fabrication involves handling the specimens at different stages, CERASET™ as liquid, thermally set solid polymer, and at its last stage as a SiCN ceramic.

Silicon wafers are used as the substrate for making thin films. The substrate is coated with a thin layer of gelatine (Bovine skin type B). This gelatine coating acts as a sacrificial layer, which will assist in removing the thermally set CERASET™ from the silicon substrate. The gelatine hardens in air within an hour, and after that CERASET™ can be applied on top of gelatine. Both the gelatine and the CERASET™ are deposited onto the silicon substrates using spin-coating and the speed of coating determines the thickness of the deposited film.

**Figure 3.**
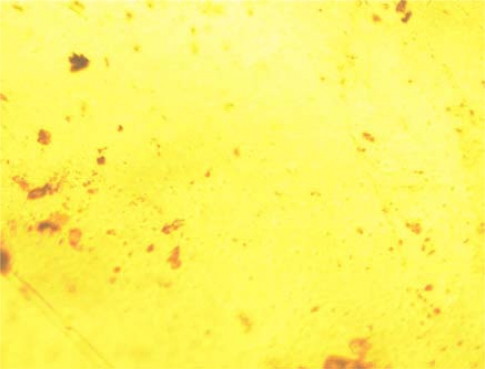
Image of the surface of polymer thin films.

The substrate is introduced to thermal setting (60 min at 120 °C in tube furnace in nitrogen gas flow) which makes the polymer soft solid and transparent. Substrate with gelatine sacrificial layer and CERASET™ polymer layer is immersed in boiled water to yield a thin film CERASET™ in the order of desired thickness. Thermoset thin film is shown in Figure 3.

**Figure 4.**
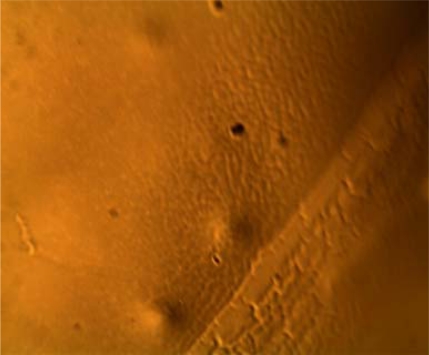
Image of the surface SiCN thin films at X 100: No visible crack appears on the surface.

The resulting thin film is crosslinked and pyrolyzed to produce a thin film SiCN ceramic. One major problem during this process is warping of the thin film. This could be minimized by sandwiching thin film obtained by thermal treatment between graphite blocks. The weight of graphite keeps the film flat and prevents wrinkling. It also does not affect the chemical reactions necessary for SiCN to form. The graphite is inert and does not attack thin films. Figure 4 shows the surface of thin film SiCN by microscopy.

**Figure 5.**
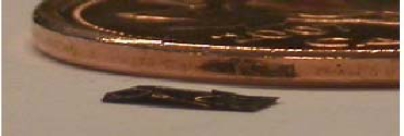
Thin film SiCN of thickness 32 micron next to a Canadian cent for size comparison.

The thickness of the SiCN specimens produced varies mostly with the rotational speed during the spin-coating process. When the CERASET™ is applied at 6,000 rpm, the specimens are very thin (∼15 μm) and subsequently are also extremely fragile. Specimens made at lower speeds between 2,000 rpm and 4,000 rpm are thicker (30–50 μm). The drying of these films without creases or folds is much easier and results in flatter, smoother specimens. These specimens are stronger and easier to handle. Some SiCN specimens are shown in Figure 5.

If the thickness of the CERASET™ layer is more than 30 μm, it is possible to burn the sacrificial gelatine layer before the pyrolysis process (at about 400–600 °C) without mechanically damaging SiCN ceramic film. Thin films (less than 30 μm thick) have to be removed by dissolving gelatine in hot water and this requires more attention while removing the gelatine layer.

**Figure 6.**
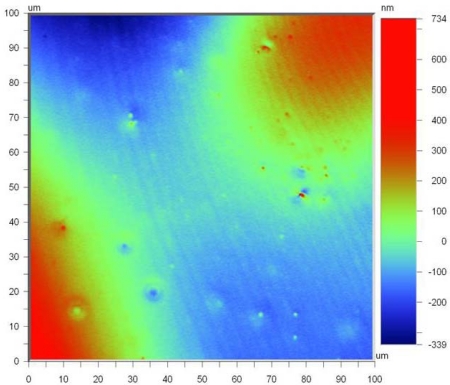
Surface roughness of thin film SiCN specimen.

Figure 6 shows the surface topography of the thin film SiCN in which the average surface roughness of the 100 × 100 μm area is 114.18 nm. The small spots shown in Figure 6 are due to the addition of dicumyl peroxide as a thermal initiator to reduce the thermosetting temperature. The peak to peak variation in the height of the asperities is around 1.07 μm. This could be considered as fairly good surface finish. The surface roughness tests were performed using optical profiler NT 1100, which has a capacity to measure with high accuracy in three dimension, using non contact surface measurements using white light interferometry.

#### 4.1.2. Thick Film SiCN

**Figure 7.**
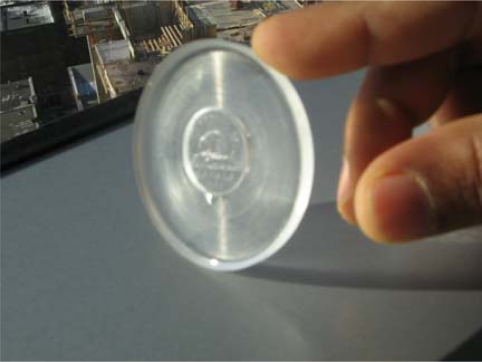
PDMS mould used to cast CERASET™.

Thick film SiCN will be used as a blocking element, which requires thickness that could reach the range of 2–3 mm. The desired thickness is much higher than the thin film and hence it is easier to handle and cast the liquid CERASET™. The idea of using soft, flexible mould is an effective way of compensating for the shrinkage as well as to adapt to the stresses developed during thermosetting of CERASET™. PDMS (polydimethylsiloxane) is obtained by mixing two chemicals (elastomer base and elastomer curing agent) and thermosetting the mixture. The ratio of elastomer base to the curing agent is made in the range of 10:1 so as to make a mould hard enough to withstand thermosetting temperature. The time for thermosetting PDMS is temperature dependent but to ensure full curing, the thermosetting is carried out for 8 hours at 60 °C. The PDMS mould made out of the procedures described above is shown in Figure 7.

**Figure 8.**
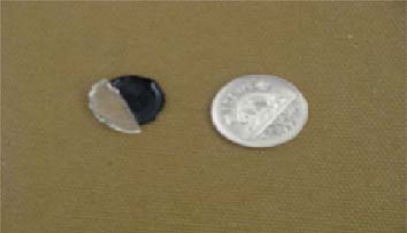
Thermally set polymer along with the ceramic.

The thermally set polymer (CERASET™) can be removed either by sacrificing the mould (dissolving in 1.0 tetrabutylammoniumfluoride (TBAF) solution in THF [23]) or by spreading aluminum as a thin sheet over the mould thereby making the mould reusable. The reusable mould is used to produce the specimens. Stresses developed and crack formation have been major problems in the fabrication of SiCN (both thin and thick films). The thermally set polymer undergoes extensive shrinking during the crosslinking and pyrolysis. Figure 8 shows the size difference between a thermally set polymer and ceramic pyrolyzed at 1,100 °C in comparison with the Canadian five cent piece of diameter 21.2 mm. It was found that there is 38.6% weight loss during transformation from thermally set polymer to SiCN ceramic and this weight loss is more significant in case of thick films than for thin films.

**Figure 9.**
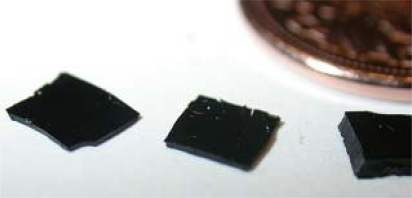
SiCN chips of different thickness.

If SiCN has to be used for different applications, it is highly desirable that SiCN could be fabricated with different thicknesses and different dimensions. Attempts have been made to produce SiCN ceramics with different thicknesses and the results are shown in Figure 9.

### 4.2. Influence of Thermosetting Temperature on SiCN Films

The thermosetting process converts liquid CERASET™ to solid polymer in the presence of heat. The thermosetting is carried out at temperatures in the range of 50–120 °C if the CERASET™ is mixed with catalyst or in the range of 200–300 °C for pure CERASET™ in the presence of nitrogen atmosphere.

The influence of thermosetting temperature on the mechanical properties of the specimen was studied for two specimens:

Specimen 1–Pure CERASET™ (without catalyst) thermally set at 240 °C
Specimen 2–Pure CERASET™ (without catalyst) thermally set at 200 °C

The specimens differ only in the thermosetting temperature (200 °C and 240 °C) with a temperature gradient of 6 °C/minute and a dwell period of 90 minutes. The solid polymer is treated under the same temperature and pressure conditions during crosslinking and pyrolysis.

The crosslinking is done at 400 °C with a dwell period of 90 minutes and a pressure of 1,800 psi (12.4 MPa). The crosslinked specimen is heated to 1,100 °C with a temperature gradient of 0.5 °C/min at a much lower pressure of 200 psi (1.38 MPa).

The hardness test reveals that specimen 2 is softer than specimen 1. The specimen 1 is tested for hardness with MST (CSEM) micro-scratch tester using the standard indentation method of the Vickers type diamond stylus. Indentation force was limited to 1 N since an excessive cracking and even specimen rupture was noticed at a higher (2N) load.

**Figure 10.**
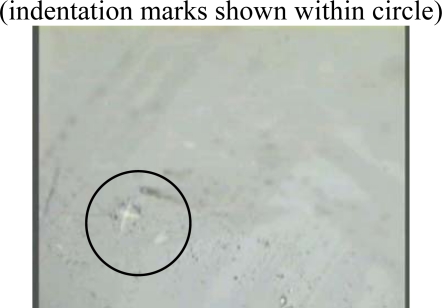
Hardness test using MST (CSEM) micro indentation tester.

The hardness of specimen 1 is estimated to be 23 GPa. Figure 10 shows the field view of the indentation done at 1 N force.

**Figure 11.**
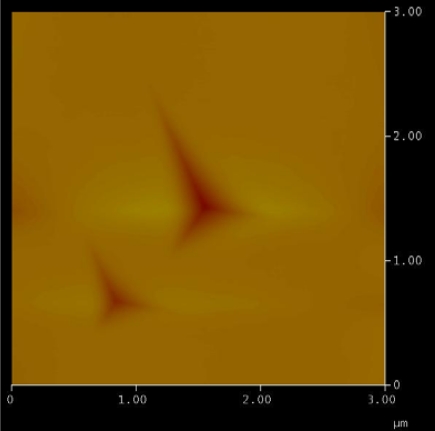
Hardness test using a hysitron nano indentator.

The hardness test on specimen 2 is done with Hysitron nanoindentor with Berkovich tip. Figure 11 shows the indentation created by the Berkovich tip at two different forces. The test is repeated at several locations on the specimen with two different loads and the average value for an indentation force of 2,500 μN and 4,000 μN. The results are presented in Table 1.

The indentation marks caused by 4,000 μN and 2,500 μN are easily recognizable in Figure 11. The average hardness value obtained is 7.5 GPa. Sufficient space between the indentations is given to record the data, as very close indentation marks may cause errors in the data obtained due to the subsequent indentations.

It is observed from these results that the thermosetting temperature has a major influence on the hardness of the SiCN made from pure CERASET™.

### 4.3. Influence of Annealing Temperature on SiCN Films

**Figure 12.**
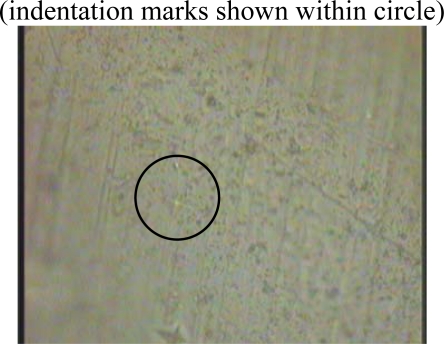
Hardness test for the specimen with highest hardness. H = 28 GPa.

CERASET™ thermally set in a condition similar to specimen 1 is crosslinked with higher isostatic pressure (2,000 psi (13.8 MPa)) and pyrolyzed and annealed at higher temperature of 1,200 °C. This resulted in a higher hardness of 28 Gpa as in Figure 12.

SiCN forms a hard solid ceramic when pyrolyzed at a temperature of 1,000 °C. After pyrolysis, the resulting SiCN is amorphous. It is highly desirable to anneal the SiCN at a higher temperature to obtain good crystal structure, higher hardness and to enable it to develop some electrical properties [26].

For studying the influence of annealing temperature two specimens were considered:

Specimen 3–CERASET™ (with catalyst) annealed to 1,500 °C
Specimen 4–CERASET™ (with catalyst) annealed to 1,200 °C

X-Ray diffraction (XRD) testing is done on specimen 3 and specimen 4, which are thermally set at 120 °C with a temperature gradient of 4 °C/minute for 90 minutes, crosslinked at 400 °C at a pressure of 1,800 psi (12.4 MPa) with a temperature gradient of 1 °C/minute, and pyrolyzed until 1,100 °C with a temperature gradient of 0.5 °C. The only difference between specimen 3 and specimen 4 is the annealing temperature.

XRD tests on specimen 4 could not provide visible peaks, as SiCN did not crystallize in specimen 4. The results from the XRD testing on specimen 3 are shown in Figure 13(a).

Analysis of the peaks in Figure 13 (b) revealed that the silicon is available in the form of SiC and β-Si_3_N_4_ and the crystallization occurs at a temperature higher than 1,200 °C. This corresponds to X-ray patterns of SiCN ceramics, pyrolyzed at 1,450 °C, and ascribed to β-Si_3_N_4_ with hexagonal structure [27]. For the specimens pyrolyzed at 1,700 °C, Li *et al.* [28] found the existence of only three peaks near 2θ = 35°. They ascribed these peaks to SiC crystallites. It implies that for the specimens pyrolyzed here above 1,600 °C the crystallization of SiC occurs, but this phase exists in our specimens, which are pyrolyzed at 1,500 °C, to less extent than Si_3_N_4_ crystallites. It is interesting to note that no peaks definitely corresponding to crystalline graphite were identified in our specimens.

The width of X-ray diffraction peaks can be used for determination of the size of SiCN crystallites. The larger the width of X-ray diffraction peak, the smaller is the size of the crystallite. The simultaneous analysis of XRD peaks at 2θ = 21°, 26°, 31°, 35°, 36°, 38°, 42°, 63°, 65° and 73° gives the average size of 40 ± 5 nm for Si_3_N_4_ crystallites in our SiCN specimens pyrolyzed at 1,500 °C. It is to be noted that formation of Si_3_ (N_1-x_C_x_)_4_ crystallites is quite possible, where a part of N ions replaced by C ions with almost the same structure as Si_3_N_4_.

**Figure 14.**
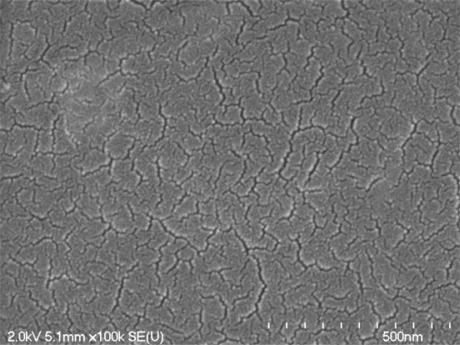
SEM image of SiCN ceramics annealed at 1,500 °C.

The overall grain size of SiCN is dependent on annealing temperature. The SEM tests were conducted on specimen 3 and 4. Specimen 4 does not show clear grains. The results reinforced the XRD results at 1,200 °C that the SiCN is still amorphous and there is no clear evidence of grain structures or boundaries. On the other hand grain structure in the range of 40–50 nm is found in specimen 3. The SEM image of specimen 3 is shown in Figure 14.

The results obtained from XRD patterns and SEM patterns support each other and gives an estimation of the grain size of SiCN ceramics annealed at 1,500 °C to be 40–50 nm. The estimations of grain size of SiCN ceramics, obtained by Fainer *et al.* [29], also support our results on grain size of SiCN ceramics annealed at 1,500 °C.

## 5. Discussion

Two different techniques for fabrication of thin and thick ceramic film plates, which can be useful for sensor details, are presented in this study. As polymer should be removed from the mold before crosslinking and pyrolysis, the usage of gelatine, which can be easily dissolved in hot water, is very effective for formation of very thin SiCN layers. Ceramic thin films with average layer thickness of 10 to 50 μm were obtained. The average surface roughness of the 100 × 100 μm area was 114.18 nm and it is fairly well for formation of the thick ceramic films.

Influence of parameters such as temperature and pressure of synthesis process were investigated and it was found that the hardest SiCN ceramics were achieved when pure CERASET™ is thermally set at a temperature of 240 °C, crosslinked at 2,000 psi (13.8 MPa) and pyrolyzed at 1,200 °C. SiCN ceramics specimens annealed below 1,200 °C were amorphous and crystallization started above this temperature. From X-ray diffraction patterns we could determine the crystalline phase of SiCN, annealed at 1,500 °C, which is mainly β-Si_3_N_4_ and the average size of these crystalline grains was determined as 40–50 nm.

We can conclude from our experiments, that amorphous, homogeneous SiCN ceramics, pyrolyzed at 1,200 °C, have better mechanical properties with a maximum hardness of 23 GPa.

## 6. Concluding Remarks

In this study, use of SiCN ceramics for building pressure sensors for high temperature applications is discussed along with the fabrication of shaped thin and thick SiCN films for use in the pressure sensor.Gelatin technique is used for thin film molding and PDMS polymer for thick film molding. The problems related to the formation of cracks on hard SiCN ceramic have also been considered. Use of a gelatine layer is suggested for fabricating thin film SiCN, which can be easily dissolved in hot water before crosslinking and pyrolysis for preparation of very thin SiCN films. This was the first time that usage of gelatin for SiCN was developed and reported. Mechanical properties of SiCN ceramics, which are required for building pressure sensors, were investigated in detail. The best mechanical properties were obtained for pure CERASET™, which was thermally set at a temperature of 240 °C, crosslinked at 2,000 psi (13.8 MPa) and pyrolyzed at 1,200 °C, for which the hardness is 23 GPa.

Following the results of this study, SiCN ceramics derived from liquid polymer polyureasilasane, can be successfully used for the design of pressure sensors of desirable shape. Experimentation on using SiCN at high temperature is in progress, and there are other issues such as data acquisition at high temperatures that has to be addressed before the experiments.

## Figures and Tables

**Figure 13.**
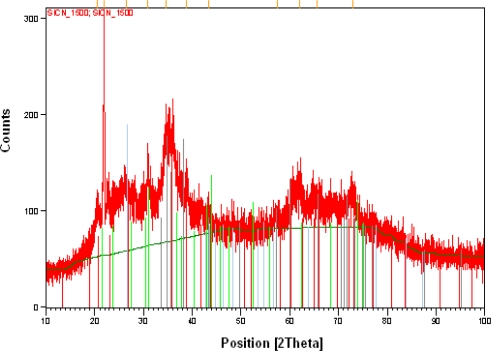
**a.** XRD peaks for a SICN annealed to a temperature of 1,500 °C.

**Figure 13.**
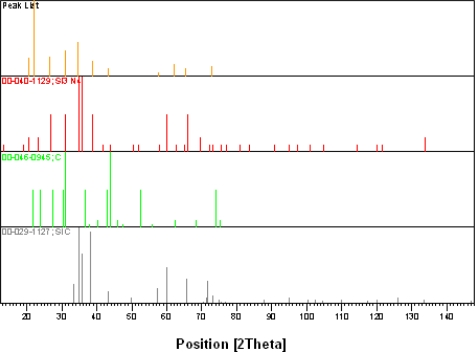
**b.** Peak list for SICN annealed to a temperature of 1,500 °C.

**Table 1.** Comparison of hardness with SiCN without catalyst at different forces.

	2,500 μN	4,000 μN
Er (GPa)	62.3	68.6
Hardness (Gpa)	7.38	7.71
Contact Depth (nm)	90.7	135.2
Contact stiffness (μN / nm)	40.3	61.8
Max force (μN)	2427.2	3908.6
Max Depth (nm)	136.2	194.9
Contact Area (nm^2^)	3.288e^5^	6.371e^5^
